# Supernumerary kidney presenting as pyonephrosis

**DOI:** 10.4103/0970-1591.56175

**Published:** 2009

**Authors:** Senthilnathan Ramasamy, Jeyakumar Paramasivam, Krishnamohan Janardhanam

**Affiliations:** Department of Pediatric Surgery, Institute of Child Health and Hospital for Children, Egmore, Chennai, India

**Keywords:** Acute nephritic syndrome, horse shoe kidney, supernumerary kidney

## Abstract

Renal anomalies constitute a majority of all congenital anomalies of urinary tract. Many anomalies warrant surgical intervention and some may not. Supernumerary kidney is an extremely rare anomaly; its association with horseshoe kidney is rare. A bizarre presentation in this patient-made preoperative diagnosis impossible. We report this extremely rare anomaly and its recognition and subsequent management.

## INTRODUCTION

Supernumerary kidney is an extremely rare renal anomaly. The association of horse shoe kidney and supernumerary kidney is exceedingly rare. We report herein one such case which presented as pyonephrosis.

## CASE REPORT

A 10 year old boy was admitted with complaints of puffiness of face, swelling of both lower limbs, and passing little quantity of urine. Patient also had fever, hematuria for two days.

The clinical examination revealed that the patient was anemic with puffy face and a bilateral pedal edema. His blood pressure was 130/100 mm Hg. Also, the abdomen examination revealed a mass in the suprapubic and umbilical regions with an undescended left testis.

Our opinion was sought, on and examination, the abdominal mass revealed a hydronephrotic pelvic kidney.

### Investigations

Ultrasonogram showed a gross hydronephrosis of a low-lying left pelvic kidney with renal pelvis crossing the midline. The contents were turbid. The right kidney collecting system was normal but showed a rotational anomaly. The Color Doppler study showed the possibility of a horseshoe kidney with abnormal vasculature.

**Figure 1:**
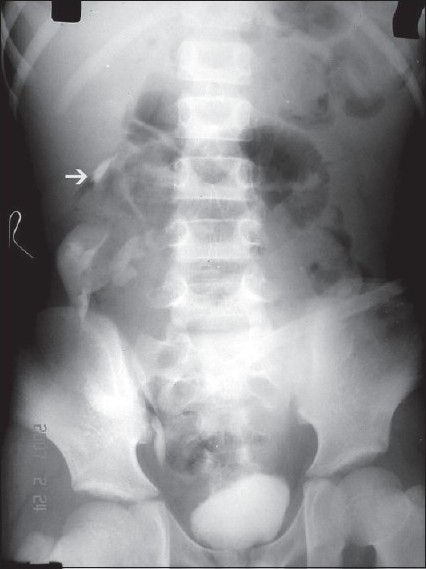
IVU. Arrow pointing to right kidney

In view of uremia, hypertension, and gross hydronephrosis a nephrostomy tube was placed and 1 1 of frank pus was drained. The general condition of the patient then improved, and the biochemical parameters and blood pressure normalized. The nephrostomy tube was draining clear urine in the ensuing days. Micturating cystourethrogram (MCU) was normal. An intravenous pyelogram (IVP) [Figure 1] was performed which revealed that the left side of the horshesoe kidney showed no function, whereas the right half functioned well with collecting system showing rotational anomaly. Cranially, the intravenous urogram (IVU) revealed a normal appearing collecting system [Figure 1]. The pediatric radiologist opined that the right half of the horseshoe kidney was displaced due to the pressure effect by the hydronephrotic left half. The figure also shows the nephrostomy tube used.

### Treatment

**Figure 2:**
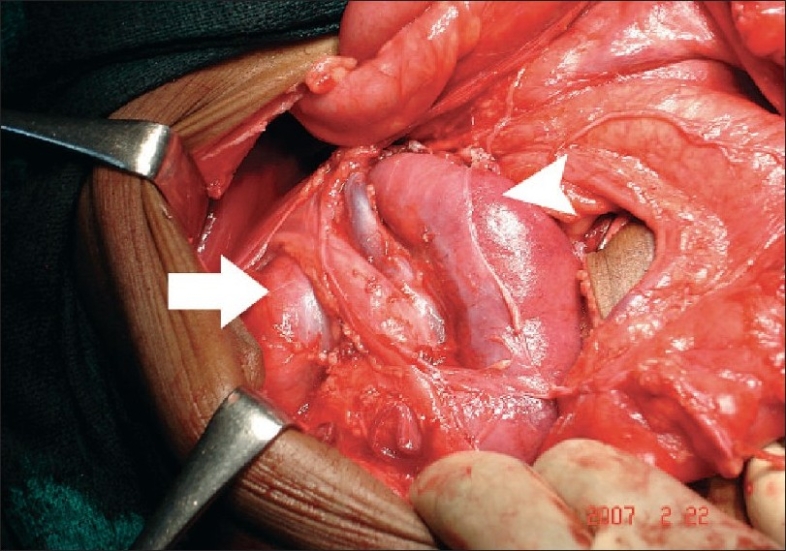
Arrow pointing to right kidney. Arrow head pointing to right half of the horse shoe kidney

We decided to explore with an open mind for a reconstructive or an ablative surgery of the left half of the horseshoe kidney. Through a midline incision, via a transperitoneal route, the kidneys were approached after reflecting ascending colon and pelvic colon. The cortex on the left part of the horseshoe kidney appeared thin and pelvi-uteretic junction (PUJ) obstruction was confirmed. The abnormal vasculature supplying the kidneys were identified and protected. The right half of the horseshoe morphology appeared bizarre with collecting system presenting on the lateral aspect of the kidney and draining to a ureter. This ureter when traced cranially led to another well-developed kidney occupying the right renal fossa [Figure 2]. This kidney had normal renal vasculature from aorta and inferior vena cava (IVC). At this juncture it was realized that the patient is having supernumerary kidney on the right side. Left nephrectomy along with isthmectomy was then completed. The postoperative period was uneventful. The histopathology of the resected left half of the horseshoe kidney showed features of glomerulonephritis.[1]

## DISCUSSION

**Figure 3:**
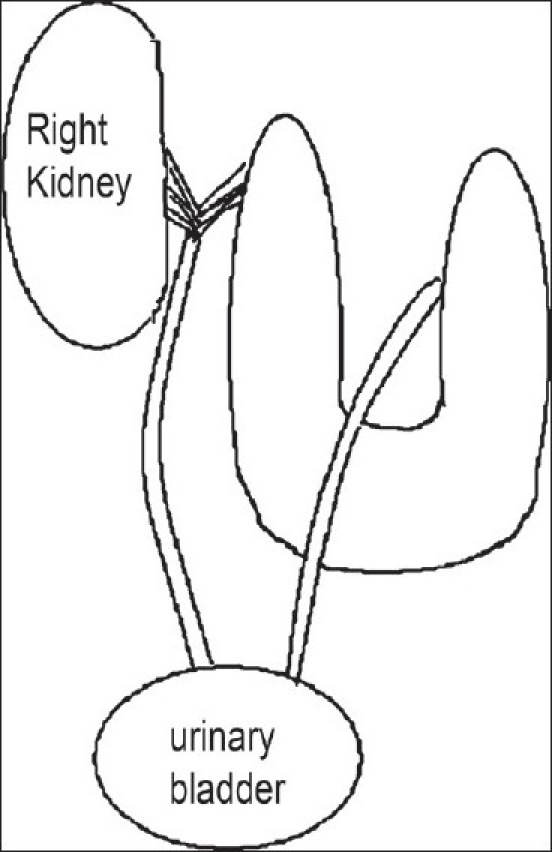
Line diagram representing the anomaly

Supernumerary kidney is an extremely rare renal anomaly, which affects both males and females equally. Since their first description in 1656, so far 80 cases have been reported.[2] There is a slight preponderance to the left side. The association of horseshoe kidney and supernumerary kidney is exceedingly rare. Various types of supernumerary kidney have been described.[2] Our case is depicted by the line diagram [Figure 3]. The supernumerary kidney is completely separate from the other kidney or may lie closely separated by thin layer of fibrous tissue. They may be cranial or caudal to the normal kidney. They have separate collecting system or may join the ureter of the dominant kidney. Supernumerary kidney usually lies in a position inferior to the normal kidney. In our case, the location of the supernumerary kidney was orthotopic which is very rare.

### Path embryology

Supernumerary kidney[3] is explained by secondary outpouching of the Wollfian duct or branching from initial ureteral bud;both induce metanephric anlage though they have separated entirely. The blood supply to the supernumerary kidney is anomalous and depends on its position in relation to the major ipsilateral kidney. In this current scenario when horseshoe kidney is associated with another well-formed kidney having normal blood supply and located in renal fossa, the metanephric blastema separates earlier and the lower anlage fuses with the left kidney at 4-6 weeks, which is a late event and this supernumerary kidney is then located in the normal renal fossa.

The sharing and predisposition of the collecting system of the horseshoe kidney on its lateral border is peculiar in this case. The collecting system of the supernumerary kidney may fuse with the collecting system of horseshoe kidney or can open separately into the bladder.

The objective of this case report is to highlight three things. First, when studying the IVU films, a normal collecting system seen in the presence of rotated collecting system, as noticed in horseshoe kidney, should strongly suspect this rare possibility of supernumerary kidney. Second, during dissection, inadvertent damage to the blood supply of the horseshoe kidney will jeopardize the collecting system of the other kidney thereby increasing morbidity, if this possibility is not borne in mind. Third, in the presence of retroperitoneal fibrosis[4] dissection can jeopardize the supernumerary kidney.

Supernumerary kidney is an extremely rare renal anomaly; may more commonly be present as urinary tract infection. Its association with horseshoe kidney is a rarity. This case presented as pyonephrosis. Despite advancements in imaging procedures, strong index of suspicion while interpreting investigations[5] will give a clue about the presence of this entity. Ignorance of this condition will lead to inadvertent dissection that will jeopardize the vascular supply leading to loss of renal units with accompanying morbidity or even mortality.
